# Host-Directed Therapy in Tuberculosis: Targeting Host Metabolism

**DOI:** 10.3389/fimmu.2020.01790

**Published:** 2020-08-13

**Authors:** Jae-Sung Kim, Ye-Ram Kim, Chul-Su Yang

**Affiliations:** ^1^Department of Molecular and Life Science, Hanyang University, Ansan, South Korea; ^2^Depatment of Bionano Technology, Hanyang University, Seoul, South Korea

**Keywords:** *Mycobacterium tuberculosis*, innate immunity, immunometabolism, host-directed therapy, inflammation

## Abstract

*Mycobacterium tuberculosis (Mtb)* has complex and intricate interactions with host immune cells. *Mtb* can survive, persist, and grow within macrophages and thereby circumvent detection by the innate immune system. Recently, the field of immunometabolism, which focuses on the link between metabolism and immune function, has provided us with an improved understanding of the role of metabolism in modulating immune function. For example, host immune cells can switch from oxidative phosphorylation to glycolysis in response to infection, a phenomenon known as the Warburg effect. In this state, immune cells are capable of amplifying production of both antimicrobial pro-inflammatory mediators that are critical for the elimination of bacteria. Also, cells undergoing the Warburg effect upregulate production of nitric oxide augment the synthesis of bioactive lipids. In this review, we describe our current understanding of the Warburg effect and discuss its role in promoting host immune responses to *Mtb*. In most settings, immune cells utilize the Warburg effect to promote inflammation and thereby eliminate invading bacteria; interestingly, *Mtb* exploits this effect to promote its own survival. A better understanding of the dynamics of metabolism within immune cells together with the specific features that contribute to the pathogenesis of tuberculosis (TB) may suggest potential host-directed therapeutic targets for promoting clearance of *Mtb* and limiting its survival *in vivo*.

## Introduction

Tuberculosis (TB) is caused by the pathogenic species, *Mycobacterium tuberculosis (Mtb)*; together with human immunodeficiency virus (HIV/AIDS) infection, TB is among the most prevalent and severe of the infectious diseases worldwide. In 2019, an estimated 10 million people developed active tuberculosis in association with 1.6 million deaths (1). Infection with *Mtb* triggers an immune response, however *Mtb* can survive and grow by circumventing the host immune detection. One of the pathological characteristics of the successful infection with *Mtb* is the formation of granulome, which are organized cellular structures that include a variety of innate and adaptive immune cells that surround the *Mtb*-infected phagocytes (2–5). During the formation of granulome, intricate host-*Mtb* interactions occur at the infectious site and this pathogen can escape various host immune responses, which ultimately prevent *Mtb* elimination by these systems. Once *Mtb* enters the host, its cell wall components and proteins are detected by Toll-like receptors (TLRs), primarily by TLR2 and TLR4. *Mtb* is engulfed by professional phagocytic cells such as a macrophage, dendritic cell (DC), or neutrophil, and becomes incorporated into the subcellular organelle formed by the fusion of the phagosome and lysosome to create the phagolysosome, however *Mtb* is able to manipulate the endocytic pathway by suppressing fusion of the phagosome containing the bacteria with lysosomes. Infected macrophages synthesize and release both inflammatory and antimicrobial genes and molecules, including interleukin (IL)-1β, IL-6, IL-12, tumor necrosis factor (TNF), inducible nitric oxide synthase/nitric oxide synthase 2 (iNOS/NOS2), and chemokines which activate both the innate and adaptive immune systems. Activated immune cells secrete protective molecules to the extracellular space to promote recruitment of other immune cells to form a granuloma (4, 6). Interestingly, endogenous proteins expressed by *Mtb* serve to perturb the formation of phagolysosome, the permitting its survival and proliferation within macrophages. For preventing excessive lung damage during *Mtb* infection, *Mtb* also elicits the production of protective factors that promote its survival including anti-inflammatory mediators such as IL-4, IL-10, IL-13, and transforming growth factor β (TGF-β) (7–9) and several human TB studies show that these factors has been shown to be increased in the active TB patients (10, 11). These immunosuppressive factors play key roles in limits effective the immune defense to *Mtb* (12, 13). *Mtb* will persist and exacerbate pathophysiological manifestations within the granulome; this will ultimately result in progression of disease and dissemination to the other hosts (5, 14). As a major focus of this disease process, mycobacterial granulome have been the subject of intense scrutiny mainly focused on mechanisms of formation, function, maintenance, and evolution.

Recently, there has been an increasing appreciation of the important relationship that exists between essential metabolism and immune cell function. Metabolic reprogramming in immune cells, a phenomenon known as immunometabolism, focuses on unique cellular functions that are essential for the immune response. During TB infection, host cells undergo profound metabolic change, which results in differential control of various cytokines and chemokines associated with inflammation, clearance, inhibition, and progression of *Mtb* infection (15, 16). Specifically, a shift in the use of pathways promoting glucose and lipid metabolism can be an important feature for directing host cell function to promote mycobacterial survival with the granulome (17). At homeostasis, cells in “resting” condition utilize oxidative phosphorylation (OXPHOS) to produce ATP from NADH and FADH2 by facilitating transfer of protons and electrons. Cells typically switch from OXPHOS to glycolysis in order to generate ATP under oxygen-depleted or hypoxic conditions (18). Similarly, glycolysis is main form of metabolism in immune cells that promote the inflammatory response in the immune system. This observation–that immune cells utilize glycolysis even in the presence of adequate concentrations of oxygen (i.e., aerobic glycolysis)– is known as the “Warburg effect.” To date, the Warburg effect has been explored primarily with respect to cancer metabolism. Although aerobic glycolysis generates fewer ATP molecules per cycle than does OXPHOS, this pathway is capable of rapid generation of ATP required by immune cells. Additionally, aerobic glycolysis requires a number of specific precursors, including nucleotides, amino acids, and lipids (19). Because metabolic reprogramming is essential for immune cell function, studies that explore this phenomenon in also provide new insight into the relationship between host immune cells and infection with *Mtb*. Furthermore, predisposing factors for TB, including diabetes, and HIV also related to immunometabolism against TB pathogenicity. Diabetes mellitus (DM) is a mainly risky factor for occurring active TB (20–22). In DM, innate immune cells undergo activation for releasing cytokines, recruiting neutrophils, upregulate T cell activation and antigen recognition (23, 24). Metabolism of DM is characterized by increasing glucose production and impairing glucose uptake. Expression of glucose transporter and glycolytic enzymes is elevated in DM (25). In DM, High glucose level increased IL-10 production, impaired macrophage phagocytic ability for promoting better milieu for survival and proliferation of TB (26, 27). Additionally, HIV is also other pathogen to be associated with pathogenicity of TB (28–30). In HIV-1-infected primary CD4^+^ T cells, glycolytic metabolism is induced with high pro-inflammatory response and increased production of virus (31, 32). Interestingly, glycolytic metabolism is regulated by HIV-1 infection in macrophage alleviated Warburg effects (33). These factors promote the activation of TB by reprogramming the metabolism.

A variety of antibiotics have been introduced for promoting eradication of *Mtb* infection, including 6–9 months courses of isoniazid, rifampicin, ethambutol, and pyrazinamide. However, the emergence of multidrug-resistant TB (MDR-TB) or extensively drug-resistant TB (XDR-TB) has become a major challenge toward designing effective treatments and for eradication of this disease (34, 35). Among the approaches to this challenge, host-directed therapy (HDT) has been introduced as a means to potentiate and to amplify the effectiveness of current treatments used for TB (36). A clear understanding of the molecular interactions between host cell metabolism and accommodations made to *Mtb* may provide new strategies to combat infection. Here we review the current understanding of the metabolic relationship between the host and the *Mtb* pathogen. We also suggest several new strategies that may enhance host metabolic pathways and thereby promote protective antimicrobial functions in the setting of TB infection.

## Metabolic Reprogramming in TB

### Warburg Effect in Immune Cells

Immune cells provide critical protection and maintain homeostasis in the mammalian host. There are currently many studies that suggest that the functions of immune cells are largely reliant on specific aspects of host metabolism. These studies, which have generated a field known as immunometabolism, have provided us for a new focus for understanding how and why immune cells exist or persist in a specific metabolic state in order to support or direct functional changes. Several recent reports suggest that different metabolic signatures have a direct impact on specific effector functions characteristic of the innate and adaptive immune systems (37). As such, among the primary functions of immune cells, there are those that generate an inflammatory response, actions typically undertaken by M1-polarized macrophages, DCs, neutrophils, and effector T cells, and those that promote an anti- inflammatory response, which include M2-polarized macrophages, as well as regulatory and memory T cells. The basic metabolic profiles of these cells differ significantly from one another. Inflammatory immune cells generate energy in the form of ATP mainly via glycolytic metabolism; by contrast, immune cells that promote anti- inflammatory activities generate ATP via oxidative phosphorylation and fatty acid oxidation (38–43). These observations have been best characterized for polarized macrophages. The predominant phenotypes of macrophages are known as M1 and M2 (44, 45). M1 macrophages, activated by lipopolysaccharide (LPS) and IFN-γ, promote pro-inflammatory and antibacterial functions in immune system, and they produce nitric oxide (NO) and reactive oxygen species (ROS) which are fundamental components of the pathways used to eradicate bacteria. The main metabolic pathway used by these cells is glycolysis, which results in rapid production of ATP via inhibition of the trichloroacetic acid (TCA) cycle and OXPHOS in mitochondria; this is a critical factor due to the fact that M1 macrophages require rapid generation of ATP to activate inflammation. By contrast, M2 macrophages promote anti-inflammatory responses and tissue repair; these cells mainly utilize OXPHOS and fatty acid oxidation in order to generate ATP; this takes place via efficient pathways localized in the mitochondria (46–51). In T cells, metabolic state is reprogrammed according to T cell subsets. Naïve T cells mainly use OXPHOS for generating energy. Upon TCR stimulation, glycolytic metabolism is upregulated for differentiating into activated T cell. Th1, TH2, and Th17 effector cells mainly depend on aerobic glycolysis. While, regulatory and memory T cells use fatty acid oxidation and OXPHOS for differentiation and functions (52, 53). Mammalian target of rapamycin (mTOR) and AKT signaling is essential for regulating metabolism of T cells and cytokine responses (54). Recently, cyclophililn D (CypD) related to necrosis is a factor for regulating metabolic state and functions in T cells (55).

Pro-inflammatory immune cells generate ATP in high concentrations via glycolysis even when functioning in aerobic conditions; the phenomenon of aerobic glycolysis is also known as the “Warburg effect” (56). Hypoxia and inflammation are inherently linked to one another; upon activation, immune cells undergo considerable metabolic reprogramming to sustain energy needs and thus switch to predominantly aerobic glycolysis. Hypoxia-induced factor 1 (HIF-1), the main mediator of the Warburg effect, is expressed in response to hypoxia and controls expression of numerous glycolytic enzymes. HIF-1 has two subunits, α and β; regulation of HIF-1 is dependent on the α subunit. Post-translational regulation of HIF- 1 is modulated via the expression and stability of HIF-1α (56–58). Members of the nuclear factor-κB (NF-κB) family of transcription factors comprise the signaling pathway that is most closely involved in Hif-1α/HIF-1A expression (59, 60). Under conditions of physiologic oxygenation, prolyl hydrolases (PHD) degrade HIF-1α and target it for proteasome-mediated degradation. Inhibiting HIF (FIH) is an aspariginyl hydroxylase that also determines the level of active HIF-1α. Overall, hypoxia-inducible genes encode proteins involved in a myriad of cellular pathways that mediate cell survival, apoptosis, erythropoiesis, angiogenesis, glucose metabolism, and that regulate acid-base balance (61). HIF-1α is expressed in primary innate immune cells, including macrophages, DCs, neutrophils, and Th17 cells. Additional roles for HIF-1α in promoting macrophage differentiation and function have also been demonstrated. Most notably, HIF-1α-mediated metabolic reprogramming plays a significant role in modulating macrophage polarization toward the M1 or M2 phenotype (62).

### Glycolysis Metabolism in TB

**Figure 1:**
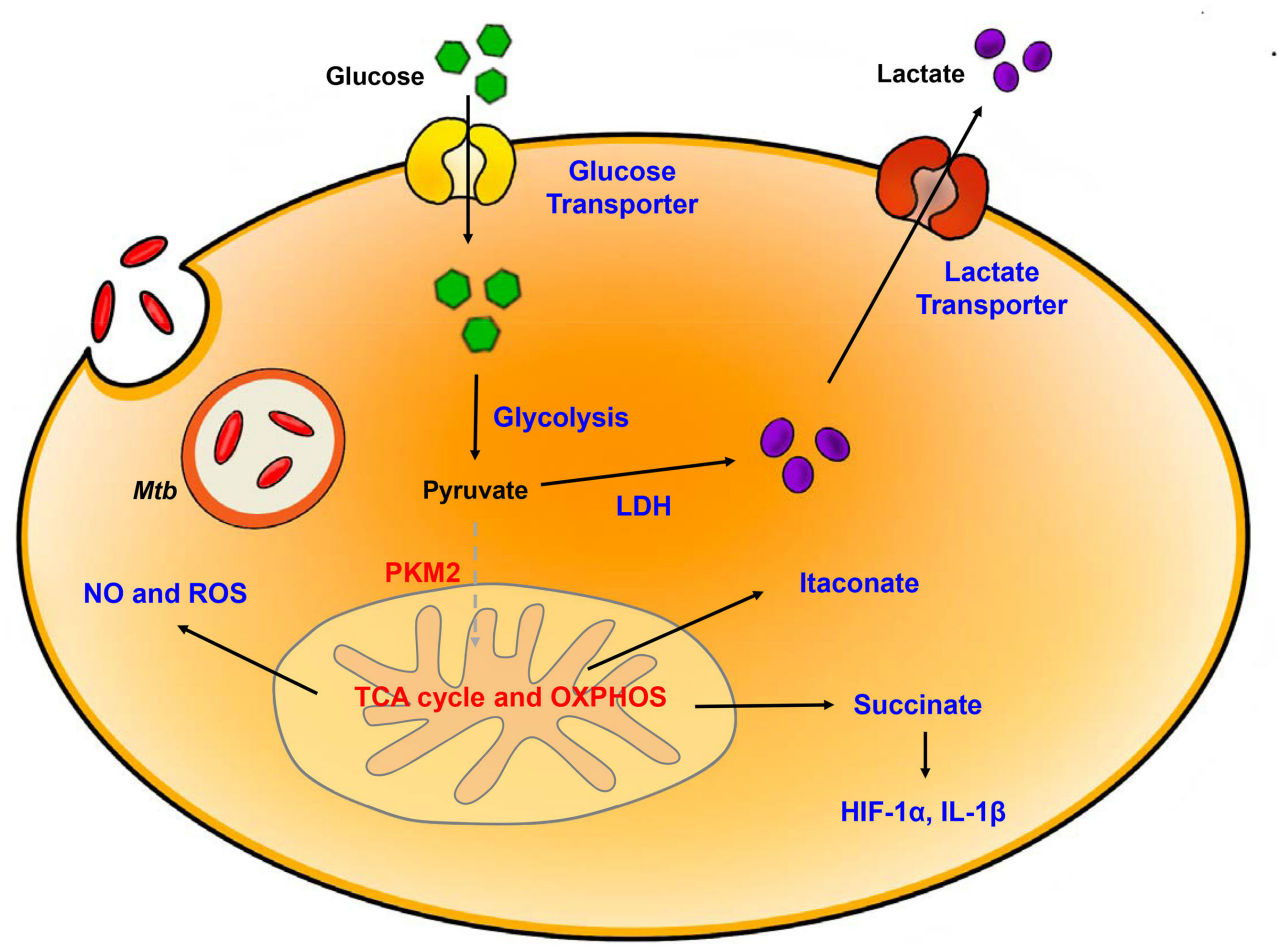
Metabolic reprogramming in *Mtb-*infected immune cells. *Mtb* infection in host is accompanied by upregulation of glycolysis and lactate production. Increased HIF-1α-induced Warburg effect enhance gene of glycolytic metabolism. In contrast, TCA cycle and oxidative phosphorylation (OXPHOS) is downregulated. Dysregulation of TCA cycle accumulates several intermediates in TCA cycle such as succinate and itaconate. Additionally, breakdown of OXPHOS increases NO and ROS level. Blue, increased expression/level; Red, decreased expression/level.

When the host is infected by bacteria, immune cells are activated; the characteristic immune response occur concomitant with a switch to glycolytic metabolism (Figure 1). Several recent studies that have focused on transcriptome data from mouse and rabbit lung as well as granulome from the lungs of TB patients suggest that the metabolic state of the TB-infected host includes modulation of glucose metabolism (63–66). The general metabolic characteristics in TB infection included enhanced expression of genes related to the Warburg effect including HIF-1α, glycolytic enzymes, the pentose phosphate pathway, and H^+^-ATPase. Additionally, ^1^H-NMR-based metabolomics profiled the increased accumulation of lactate due to the increased levels of glycolysis in the lungs of *Mtb*-infected mice (67). Likewise, host immune cells responded to *Mtb* infection with increased expression of pro- inflammatory and antimicrobial-related genes associated with the Warburg effect. These results highlighted the importance of metabolic reprogramming due to glycolysis and its relationship to protection against *Mtb* infection. Furthermore, analysis of the transcriptomes of bone marrow-derived macrophages (BMDM) infected with one of two clinical strains of *Mtb* (the immunogenic strain CDC1551 or the hypervirulent strain HN878) included elevated levels of expression of genes associated with the Warburg effect. Given that these two clinical strains are known for differential activation of immune responses during the course of BMDM infection, different metabolic responses were anticipated (64). Interestingly, BMDMs infected with each strain promoted upregulation of genes encoding enzymes associated with the Warburg effect together with HIF-1α-associated signaling, although specific differences were observed. Of note, at 6 h post-infection, the induction of the gene encoding 6-phosphofructo-2-kinase/fructose-2,6-biphosphatase 3 (PFKFB3) a member of the of phosphofructokinase (PFK)-2 family, was more prominent in CDC1551-infected BMDMs (65). *Pfkfb3* has the highest activity among the PFK-2 members, and fructose-2,6-diphosphate (F-2,6-BP), which is the product of Pfkfb3-mediated phosphorylation, is an essential component promoting regulation of glycolysis (68). CDC1551-infected BMDMs in a state of elevated glycolysis respond with a vigorous early pro-inflammatory response. By contrast, relatively limited activation of the Warburg effect together with high levels of glucose uptake were observed in response to *Mtb*. Furthermore, HN878-infection of BMDMs may result in dysregulated host cell lipid metabolism. Specifically, one study compared gene expression in response to *Mtb* H37Ra or H37Rv infection of human alveolar macrophage revealed strain-specific differences. Gene expression associated with inflammation, general metabolism, and lipid metabolism was downregulated in H37Rv infected macrophages (69). As suggested by the responses to infection with HN878, a virulent strain can have an impact on host metabolism gene by downregulating inflammatory responses that results in diminished the inflammation and prolonged *Mtb* survival. Another study compared the metabolic states elicited by macrophage challenge with *Mtb*, with the vaccine strain *M. bovis* BCG or with killed *Mtb*. Each strain promoted a unique pattern of energy modulation, as determined by XF (extracellular flux) analysis. Total metabolism in response to challenge with live *Mtb* including glucose utilization and OXPHOS is lower than that observed in response to BCG or dead *Mtb* (70). Also, CD8^+^ T cell showed similar results in *Mtb* or BCG infection. Through RNA-seq, glycolytic metabolism is upregulated by challenging *Mtb* in early and late phase. Surprisingly, *Mtb* triggered mitochondrial dysfunction, which downregulates OXPHOS metabolism, while upregulates mtROS, but metabolism is recovered against BCG (71). Thus, infection with live, virulent *Mtb* decelerated the shift to glycolytic and OXPHOS bioenergetics, and thereby limited the development of inflammatory effector functions.

**Figure 2:**
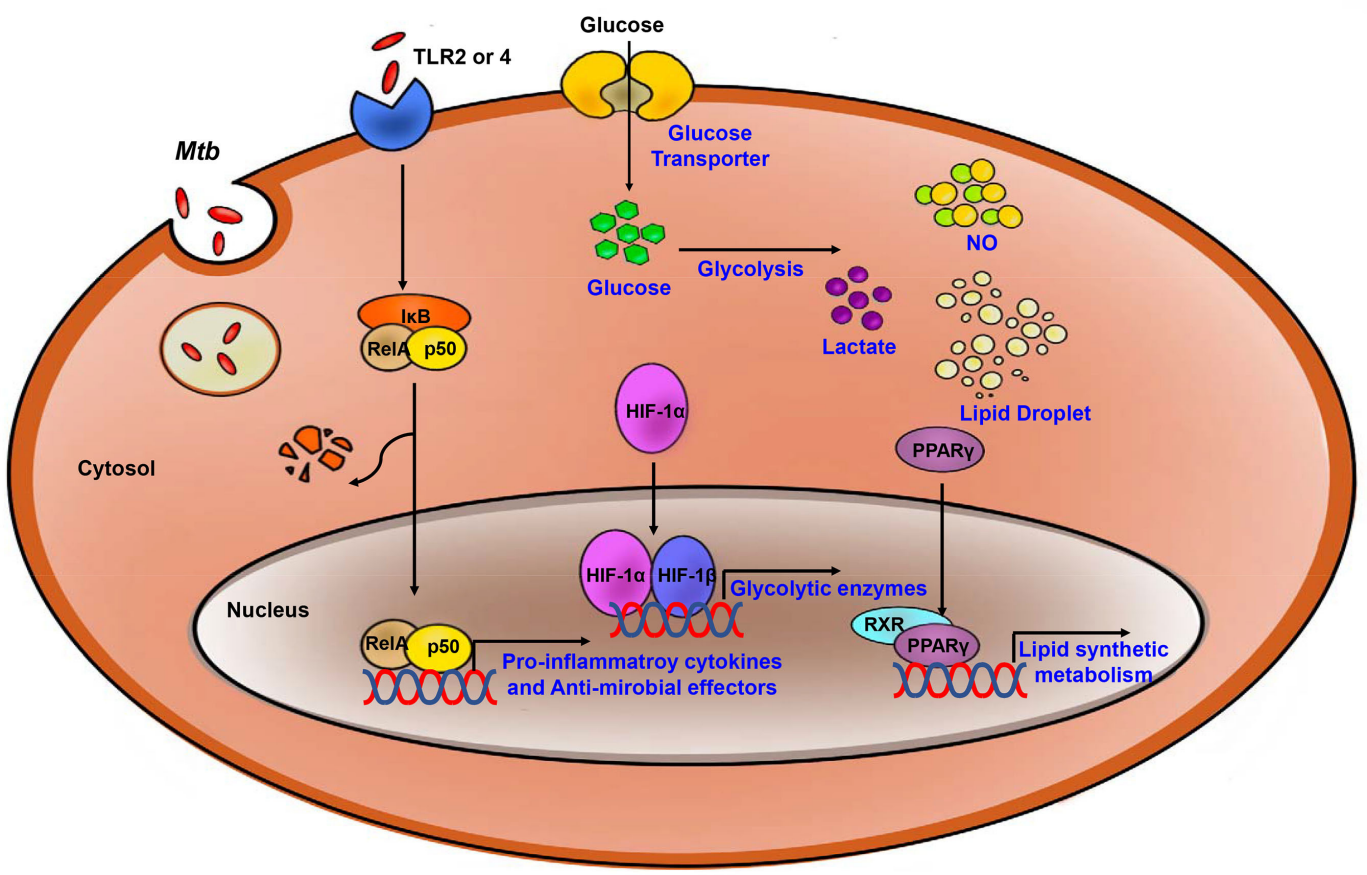
Process of the Immune response and metabolic reprogramming in *Mtb-* infected immune cells. After *Mtb* infection, inflammatory signaling is activated by TLR2 or 4. Also, Metabolism is switch to aerobic glycolysis mediated by HIF-1α which upregulates glycolytic enzymes. Increased glycolysis related to upregulate pro-inflammatory cytokines and anti-microbial effectors. PPARγ upregulates lipid synthetic gene for formation of lipid droplet which is exploited by *Mtb* for survival and growth. Blue, increased expression/level.

The switch to glycolytic metabolism resulted in the accumulation of several TCA intermediates that themselves function as a metabolic signal to link metabolism and immunity (Figure 2). Succinate, a prominent TCA intermediate, drives IL-1β production, inhibits the production of anti-inflammatory cytokines, and enhances HIF-1α activity by inhibiting HIF-1α prolyl hydrolases (72–74). The succinate-induced pro-inflammatory response is directly dependent on the activity of succinate dehydrogenase (SDH). Inhibition of SDH activity via hydrolysis of dimethyl malonate to produce malonate, results in an attenuation of the activity of LPS-induced IL-1β, and likewise a boost in IL-10 production in BMDMs generated from C57BL/6 mice (75). In *Mtb*-infected murine macrophages, *Sdh* expression is downregulated; this leads to the induction of HIF-1α, the Warburg effect, and characteristic pro-inflammatory responses (76). Itaconate, a metabolite derived from the TCA cycle intermediate cis-aconitate, also regulates SDH activity in C57BL/6 BMDMs (77, 78). Breakdown of TCA cycle results in downregulation of mitochondrial isocitrate dehydrogenase (*Idh*)2 immediately following formation of itaconate. Aconitate decarboxylase 1 (*ACOD1*), is also known as immune-responsive gene (Irg)1; production of this mediator is related to generation of itaconate. *ACOD1* is upregulated in *Mtb*-infected murine macrophages and lung tissue. Itaconate has antimicrobial functions via its capacity to inhibit isocitrate lyase, the essential enzyme in the glyoxylate shunt that is critical for bacterial growth. Itaconate inhibits SDH activity which results in the accumulation of succinate. Additionally, itaconate modulates pro- inflammatory responses in macrophage; *Irg1*^−/−^ BMDMs from C57BL/6 mice maintain higher HIF-1α mRNA and protein levels, and produce more pro-inflammatory cytokines and antimicrobial factors including IL-6, IL-12, IL-1β, and NO in response to lipopolysaccharide (LPS)-mediated activation (79). Thus, itaconate may be a critical link between the Warburg effect induced by *Mtb* infection, and the generation of anti-inflammatory responses to prevent damage to host cells.

Upregulated expression of HIF-1α, the enhanced Warburg effect, and the antimicrobial response to *Mtb* infection of host immune cells are all linked to the actions of the glycolytic regulatory protein, pyruvate kinase M2 (PKM2). Expression of PKM2, one of the two Pkm/PKM gene products, is upregulated in response to macrophage activation. In the cytoplasm, PKM2 maintains an enzymatically inactive state via its phosphorylation; the PKM2 dimer is transferred into the nucleus where it interacts with HIF-1α to activate target genes, including those encoding glycolytic enzymes and IL- 1β. In LPS-activated macrophages, small molecules such as TEPP-46 modulate PKM2 activation by preventing PKM2 translocation into the nucleus; consequently, results in a diminished Warburg effect and limited production of IL-1β. Inhibition of PKM2 translocation also promotes production of IL-10 and a decreased antimicrobial response in an *S. typhimurinum* infection model (80). In transcriptome analysis studies, upregulation of Pkm2/PKM2 was detected in *Mtb*- infected murine macrophages and in mouse lung tissue (65). These results suggest that, similar to itaconate, PKM2 promotes the HIF-1α-mediated Warburg effect and the associated antimicrobial response during *Mtb* infection. CypD, mitochondrial matrix protein, is regulator of metabolism in *Mtb* infection via upregulating mtROS in T cells. CypD-deficient T cells showed higher OXPHOS than wild-type T cells and more susceptible to *Mtb* (55).

In summary, metabolism in *Mtb*-infected host cells undergoes a switch from OXPHOS to glycolysis and generates a Warburg effect. The HIF-1α induced Warburg effect in the setting of TB infection plays an essential role in promoting upregulation of pro-inflammatory cytokine and antimicrobial effector gene expression, both factors underlying the acute immune response. However, host immune responses were different depending on the virulence or avirulence of the *Mtb*-infecting strain. How and why immune responses are modulated by different strains of *Mtb* are not fully understood.

### Arginine Metabolism in TB

Arginine, the key substrate for production of NO and other reactive nitrogen species, and also serves as a substrate for arginase. Arginine plays a distinct role in the host immune response. iNOS promotes one pathway that results in the generation of NO; the other pathway is via the arginase-mediated production of ornithine (16). iNOS is one of three NO synthase enzymes and the major isoform involved in immune cell functions. iNOS is inducible in immune cells, and is a prominent antimicrobial effector molecule produced by activated macrophages (81). The balance of arginine metabolism between the two competing pathways constitutes an important regulatory mechanism that modulates the polarization states of M1 and M2 macrophages. In M1 macrophages, arginine is in demand for protein synthesis, for production of NO, and for its antimicrobial roles; by contrast, in M2 macrophages, arginine is used for production of polyamines and proline. The iNOS pathway is in direct competition with the arginase pathway (82, 83). Two arginase isoforms exist in the cells. Cytosolic arginase ARG1 and mitochondrial arginase ARG2 are encoded by different genes and have different subcellular distributions (84, 85). ARG1 is mainly detected in murine myeloid cells, DCs, and granulocytes. ARG1 inhibits NO production from iNOS/NOS2 which is among the mechanisms used by *Mtb* for immune evasion. *Mtb*-infected *Arg1* conditional gene-deleted mice were characterized with a diminished bacterial burden; Arg1-deficient macrophages were more capable of killing *Mtb* compared to their wild-type counterparts (86). ARG1 and iNOS are distributed in distinct patterns in human TB-associated granulome; expression of iNOS was highest in the central region, and ARG1 was more prominent at the periphery (87). The role of ARG1 in mediating immune cell function is directly dependent on the stage of *Mtb* infection. At initial stages of infection, the Mtb pathogen takes advantage of ARG1 activity by limiting macrophage immunity via competition with iNOS/NOS2. During the late stages of infection, ARG1 contributes to control of prolonged hyperinflammation; ARG1 also plays a role in regulating the progression of lung immunopathology in *Mtb*-infected, Nos2-deficient mice (87).

### Lipid Metabolism in TB

Once glycolytic metabolism has been activated, the genes encoding pro- inflammatory mediators are synthesized, together with the synthesis of fatty acids and phospholipids. The TCA cycle and OXPHOS are inhibited, and several intermediates of the TCA cycle accumulate *in situ* (88). Similar to what has been observed for glucose metabolism, including the TCA cycle and OXPHOS, host lipid metabolism is also regulated in *Mtb* infection (Figure 2). There are master regulators that mediate lipid metabolism including the peroxisome proliferator-activated receptors (PPARs), liver X receptor (LXR), sterol regulatory element binding proteins (SREBPs) and HIF (89–93). These factors work together to regulate processes including fatty acid uptake, lipid synthesis, the activities of lipolytic enzymes, and lipid droplet (LD) biogenesis (94). The activation of TLR signaling upregulates expression of several enzymes that promote synthesis of triglycerides and/or cholesterol ester, including fatty acid synthase (FASN), diacylglycerol O- acyltransferases (DGAT-1 and DGAT-2), and acyl-CoA:cholesterol O-acyltransferases (ACAT1 and ACAT2) (95–97). During lipid accumulation, increased expression of lipid uptake and transport-related genes is observed, and expression of genes involved in lipolysis is decreased. Perilipin-2 (Plin2) and Perilipin-3 (Plin3) are the main structural proteins of LDs that serve to promote lipid accumulation (96, 98, 99). These proteins are essential for the biogenesis and assembly of LDs (100).

PPARs are members of the ligand-activated transcription factor family (101). PPARs can have a direct impact on LD formation via the regulation of Plin2 expression. PPARs also regulate proteins associated with *de novo* lipogenesis, including fatty acid synthase and gene regulatory factors LXR and SREBPs (94). PPAR-γ is important for regulating lipid and glucose metabolism and other cellular process including inflammation (102). Host immune cells which are infected by *Mtb* exhibit increased PPAR-γ gene expression; this results in downregulation of NF-κB signaling and increases in production of prostaglandin (PG) E2; overall, this results in suppression of pro- inflammatory cytokines and Th1 responses (103, 104). Increased PPAR-γ expression in *Mtb*-infected macrophages is also associated with LD formation (105). Formation of LDs is critical for bacterial survival; the accumulated lipids in these infected cells provide nutrients and promote bacterial growth in host. Additionally, infection with *M. bovis* BCG results in upregulated expression and activation of PPAR-γ and the induction of lipid-loaded macrophages. In BCG-infected TLR2-deficient mice, production of TNF-α undergoes significant downregulation (104, 106). Taken together, these findings suggest that PPAR-γ accelerates intracellular lipid accumulation by modulating the expression of genes that modulate lipid absorption as well as those that promote fatty acid synthesis in response to *Mtb* infection.

PPAR-α is another isoform of the PPAR family. It is a transcription factor that modulates the expression of several genes involved in lipid oxidation and glucose metabolism (107). PPAR-α enhances fatty acid oxidation and ketogenesis while inhibiting fatty acid synthesis and glycolysis (108). As such, activation of PPAR-α may prevent lipid accumulation in *Mtb*-infected cells. PPAR-α activation also results in the upregulation of transcription factor EB (TFEB) and promotes host innate immunity and autophagy against *Mtb* infection. The induction of TFEB also promotes lipid catabolism which inhibited intracellular growth of *Mtb* growth in bone marrow-derived macrophages (109).

## Metabolic HDT in TB

In recent years many researchers have demonstrated that changes in dynamic immunometabolism take place in response to infection with microbes; as such, studies focused on immunometabolism are important so as to provide a larger understanding of their role in promoting pathogenesis in host (110). Current clinical trials have limitations with respect to the elimination of *Mtb* infection, including the need for long-term use, severe side effects, and the emergence of drug-resistant strains (111). As noted above, *Mtb* infection can induce a Warburg effect in host immune cells, similar to that described in tumor tissue (65). *Mtb* exploits host metabolism in order to escape immune surveillance and modulates various responses to subvert their activities toward promoting its survival and longevity. We expect HDT to be a clinically-feasible approach toward readjusting uncontrolled immune responses in patients with infectious disorders. We discuss HDT drugs currently in use or under development that target host metabolism. We will also suggest novel candidate HDT pathways and agents that might be effective toward eradicating *Mtb* (Table 1).

**Table 1:** Host-directed therapies that regulate host metabolism in TB.

**HDT in glucose metabolism**			
**Name**	**Target**	**Result**	**References**
2-deoxyglucose	Hexokinase	Inhibition of glycolysis Suppression of IL-1β	(73, 112)
3-bromopyruvate	Hexokinase	Inhibition of glycolysis	(113)
Ritonavir	Glucose transporter	Inhibition of glycolysis	(114)
Dichloroacetate	Pyruvate dehydrogenase kinase	Inhibition of glycolysis	(115)
FX11	Lactate dehydrogenase	Inhibition of glycolysis Downregulation of cytokines and iNOS	(116)
TEPP46	Pyruvate kinase M2	Inhibition of HIF-1α Suppression of IL-1β	(80)
Rapamycin	mTOR	Inhibition of glycolysis Upregulation of antimicrobial effect	(117, 118)
Loperamide	mTOR	Inhibition of glycolysis Upregulation of antimicrobial effect	(119)
**HDT in lipid metabolism**			
Metformin	AMP kinase	Increased fatty acid oxidation. Inhibition antibacterial activity Reduced gene of inflammation	(120, 121)
AICAR	AMP kinase	Increased antibacterial activity Induced mitochondrial biogenesis and energy metabolism Inhibition of lipid synthesis	(122)
C75	Fatty acid synthase	Inhibition of fatty acid synthesis Reduced the inflammation and oxidative stress Switch M2 to M1 Downregulation of NLRP3 inflammasom	(123–125)
Cerulenin	Fatty acid synthase	Inhibition of fatty acid synthesis Downregulation of NLRP3 inflammasome	(125)
GW9662	PPARγ	Modulation of lipid metabolism, inflammation and pathogenesis of bacteria	(95)
Sirtuins	PGC-1α	Inhibition of NF-κB signaling and proinflammatory response Upregulation of fatty acid oxidation and anti-inflammation	(76, 126–128)

*HDT in glucose metabolism HDT in lipid metabolism*.

### HDT in Glucose Metabolism

In TB infection, metabolism switches to glycolysis in order to protect the host against early-phase *Mtb* responses. HIF-1-dependent glycolysis promotes various immune effector functions including production and release of pro-inflammatory cytokines and NO. As noted earlier, virulent *Mtb* perturbs the glycolytic metabolism and thereby inhibits antimicrobial functions. These results suggest metabolic reprogramming to aerobic glycolysis is essential component of the anti-TB response. On the other hand, persistent inflammation can result in hyperinflammation and ultimately damage host cells and tissues. Among the featured mechanisms of HDT in TB, there is a focus on inhibition of glycolysis as well as modulation of mTOR and AMP-activated protein kinase (AMPK) pathways. For example, 2-deoxyglucose (2-DG) and 3-bromopyruvate suppress activity of hexokinase which is a critical enzyme that catalyzes the first step of glycolysis (113). In LPS-activated macrophages, 2-DG suppresses the production of IL-1β and results in the accumulation of succinate (73). Additionally, LPS-induced acute lung injury is reduced by 2-DG-dependent inhibition of glycolysis (112). Among others under consideration is the HIV-protease inhibitor, ritonavir, which is an antagonist of glucose transporters (114), dichloroacetate, an inhibitor of pyruvate dehydrogenase kinase (115), and FX11, a specific inhibitor of lactate dehydrogenase. In LPS-activated RAW 264.7 mouse macrophages, FX11-mediated inhibition of lactate dehydrogenase resulted in the downregulation of cytokine and iNOS production (116). Likewise, TEPP46 is small molecule that inhibits the activity of pyruvate kinase M2; this inhibitor attenuates activation of PKM2 in LPS-induced macrophage *in vivo* and results in suppression of IL-1β production (80).

Induction of autophagy can be potential defense strategy used by cells to eradicate *Mtb* infection. The enzyme, mTOR kinase, negatively regulates autophagy; as such, mTOR kinase inhibitors may be potent candidates for HDT for the elimination of *Mtb* infection. Other mTOR inhibitors including rapamycin and torin serve to limit the increased levels of lactate detected in *Mtb*-infected macrophages (54). Rapamycin-mediated activation of autophagy results in acidification of mycobacterial phagosomes and thus decreased survival of BCG (117). Loperamide induces mTOR-independent autophagy and likewise controls intracellular *Mtb* burden in lung macrophages (119). However, the use of these inhibitors has several limitations. For example, rapamycin-induced autophagy resulted in enhanced intracellular bacterial replication in HIV/H37Rv co-infected cells (118). Therefore, pharmacological induction of autophagy should be carefully evaluated among the candidate drugs to be used for HDT.

### HDT in Lipid Metabolism

*Mtb* exploits host lipid or fatty acid metabolism to promote its own survival and growth. Foamy macrophages are recruited to granulome where and are included in the barrier that forms around *Mtb*-infected phagocytic cells to which they provide support and nutrition. Toward this end, infection with *Mtb* induces the synthesis of LDs and fatty acids in host cell. Targeting the lipid synthesis may be a good strategy for initial HDT with the goal of eliminating *Mtb*. 5' AMPK is a highly conserved master regulator which can restore the energy balance by shifting cellular metabolism from one that consumes ATP to a catabolic mechanism that generates ATP (129). AMPK and other metabolic energy sensors are critical in maintaining various functions of *Mtb*-infected host immune cells, including autophagy, fatty acid β- oxidation, and metabolic reprogramming; the AMPK pathway also plays multi-faceted roles in promoting host defense against viral and bacterial infection. As such, molecules that are targeted by AMPK-targeted are considered to be effective adjuvant agents used to combat *Mtb* infection (130, 131). Metformin, a drug that is clinically-approved for the treatment of type 2 diabetes functions by activating the AMPK-mediated signaling pathway (121). Treatment with metformin can limit intracellular *Mtb* growth in macrophages via induction mitochondrial ROS and can thereby reduce activation of inflammatory-related gene expression. Also, metformin shows some synergy with conventional anti-TB drugs, including isoniazid or ethionamide when evaluated in *Mtb*-infected mice. Metformin treatment also decreases the incidence of latent TB (120). AICAR (5-aminoimidazole-4-carboxamide-1-β-D-ribofuranoside) is another agent that activates AMPK; AICAR activates autophagy pathways in macrophages and thus promotes antibacterial activity against *Mtb*. AICAR-mediated AMPK-activation also results in the activation of the PPARGC1 (peroxisome proliferator-activated receptor gamma, coactivator 1) pathway; this latter pathway regulates mitochondrial biogenesis and energy metabolism in macrophages and in *Drosophila melanogaster* infected with *M. marinum* (122).

Factors that suppress lipid synthesis can limit inflammation and balance the inflammatory state of the host. Among several candidate molecules, C75 and cerulenin inhibit fatty acid synthase. C75 effectively lowers free fatty acid accumulation in mice with sepsis and limits inflammation and oxidative stress (123). Additionally, C75-mediated inhibition of lipid-derived droplet formation results in a switch from M2 to M1 macrophage polarization, resulting in enhanced production of both ROS and NO generation (124). Additionally, inhibition of fatty acid synthase by C75 and cerulenin results in downregulated uncoupling protein (UCP2)- mediated NLRP3 inflammasome activation (125). GW9662, an antagonist of PPARγ, acts as a key modulator of lipid metabolism, inflammation, and pathogenesis in BCG-infected macrophages; this result suggests that regulation of lipid metabolism may be a strong potential host target for novel TB therapy (91). Likewise, sirtuins (SIRTs) have been recognized as potential targets for anti-TB therapeutics. Sirtuins are enzymes with deacetylase activity that modulate cellular process by inhibiting NF-κB signaling; this results in a downregulation of the pro-inflammatory response and upregulation of fatty acid oxidation and anti- inflammatory response by targeting Peroxisome proliferator-activated receptor gamma coactivator 1-alpha (PGC-1α) (126, 127). SIRT1 expression is diminished in *Mtb*-infected THP-1 macrophages and in whole mouse lung tissue. SIRT1 promotes inflammatory resolution by downregulating the expression of the RelA/p65 unit of NF-κB (128). SIRT6 also suppress pro-inflammatory and antimicrobial responses at the early stages of *Mtb* infection (76).

## Conclusion

Immunometabolism is among the critical features that define the intimate relationship between host and the *Mtb* pathogen; a clear understanding of these interactions will be essential for limiting the progression of the TB. Metabolic reprogramming from OXPHOS to glycolysis in *Mtb* infection results in the upregulated expression of numerous pro-inflammatory cytokines and antimicrobial effector molecules. Further investigation will be needed in order to understand more fully the relationship between *Mtb* and host metabolism. How and when *Mtb* exploit the host metabolism is not clearly understood at this time; clarification will be critical in order to identify the most appropriate candidates for HDT. Among those currently under consideration is *Mtb*-mediated modulation of glucose and/or lipid metabolism. Glucose metabolism might be targeted at the early stage, which would ultimately provide a boost to the Warburg effect. Thus, more efficient elimination of *Mtb* bacteria; by contrast, targeting glucose metabolism at a later stage may result in a much needed- alleviation of hyperinflammation. A better understanding of metabolic reprogramming in TB will provide further insights toward novel therapeutic strategies.

## Author Contributions

J-SK, Y-RK, and C-SY designed, conceptualized, and wrote the manuscript. All authors contributed to the article and approved the submitted version.

## Conflict of Interest

The authors declare that the research was conducted in the absence of any commercial or financial relationships that could be construed as a potential conflict of interest.

## Abbreviations

*Mtb*: *Mycobacterium tuberculosis*
TB: Tuberculosis
HDT: Host-directed target
TLRs: Toll-like receptors
DC: Dendritic cell
IL: Interleukin
TNF: Tumor necrosis factor
iNOS/NOS2: inducible nitric oxide synthase/nitric oxide synthase 2
TGF-β: Transforming growth factor β
OXPHOS: Oxidative phosphorylation
DM: Diabetes mellitus
MDR-TB: Multidrug-resistant TB
XDR-TB: Extensively drug-resistance TB
NO: Nitric oxide
ROS: Reactive oxygen species
HIF-1: Hypoxia-induced factor 1
NF-κB: Nuclear factor-κB
CypD: Cyclophililn D
PHD: Prolyl hydrolases
FIH: Factor inhibiting HIF
PFKFB3: 6-phosphofructo-2-kinase/fructose-2,6-biphosphatase 3
F-2,6-BP: Fructose-2,6-diphosphate
SDH: Succinate dehydrogenase
LPS: Lipopolysaccharide
ACOD1: Aconitate decarboxylase 1
Irg1: Immune-responsive gene1
PKM2: Pyruvate kinase M2
ARG: Arginase
PPARs: Peroxisome proliferator-activated receptors
LXR: Liver X receptor
SREBPs: Sterol regulatory element-binding proteins
LD: Lipid droplet
FASN: Fatty acid synthase
DGAT: Diacylglycerol O-acyltransferase
ACAT: Acyl-CoA:cholesterol O- acyltransferase
Plin: Perilipin
TFEB: Transcription factor EB
mTOR: Mammal target of rapamycin
AMPK: AMP-activated protein kinase
2-DG: 2-deoxyglucose
PPARGC1: Peroxisome proliferator-activated receptor gamma, coactivator 1
AICAR: 5-aminoimidazole-4-carboxamide-1-β-D-ribofuranoside
UCP2: Mitochondrial uncoupling protein 2
SIRTs: Sirtuins
PGC-1α: Peroxisome proliferator-activated receptor gamma coactivator 1-alpha.
